# Automating reflectance confocal microscopy image analysis for dermatological research: a review

**DOI:** 10.1117/1.JBO.27.7.070902

**Published:** 2022-07-25

**Authors:** Imane Lboukili, Georgios Stamatas, Xavier Descombes

**Affiliations:** a Johnson & Johnson Santé Beauté France, Paris, France; b Université Côte d’Azur, INRIA, I3S/CNRS, Nice, Antibes, France

**Keywords:** reflectance confocal microscopy, skin, epidermis, machine learning, epidermal layer classification, dermal–epidermal junction delineation, cell demarcation, skin lesions

## Abstract

**Significance:**

Reflectance confocal microscopy (RCM) is a noninvasive, *in vivo* technology that offers near histopathological resolution at the cellular level. It is useful in the study of phenomena for which obtaining a biopsy is impractical or would cause unnecessary tissue damage and trauma to the patient.

**Aim:**

This review covers the use of RCM in the study of skin and the use of machine learning to automate information extraction. It has two goals: (1) an overview of information provided by RCM on skin structure and how it changes over time in response to stimuli and in disease and (2) an overview of machine learning approaches developed to automate the extraction of key morphological features from RCM images.

**Approach:**

A PubMed search was conducted with additional literature obtained from references lists.

**Results:**

The application of RCM as an *in vivo* tool in dermatological research and the biologically relevant information derived from it are presented. Algorithms for image classification to epidermal layers, delineation of the dermal–epidermal junction, classification of skin lesions, and demarcation of individual cells within an image, all important factors in the makeup of the skin barrier, were reviewed. Application of image analysis methods in RCM is hindered by low image quality due to noise and/or poor contrast. Use of supervised machine learning is limited by time-consuming manual labeling of RCM images.

**Conclusions:**

RCM has great potential in the study of skin structures. The use of artificial intelligence could enable an easier, more reproducible, precise, and rigorous study of RCM images for the understanding of skin structures, skin barrier, and skin inflammation and lesions. Although several attempts have been made, further work is still needed to provide a definite gold standard and overcome issues related to image quality, limited labeled datasets, and lack of phenotype variability in available databases.

## 1 Introduction to Reflectance Confocal Microscopy

For a long time, biopsies followed by histological and microscopic analysis were the gold standard in studying skin structure. Unfortunately, biopsies are invasive and lead to local inflammation due to the damaged cells, which may alter the studied sample and hinder the study of healthy skin. They can also be traumatizing for the patient, particularly when done repeatedly, e.g., for skin cancer monitoring. In certain cases, biopsies can raise ethical questions, such as for cosmetic testing^1^ or for the study of healthy infant skin. Therefore, alternatives to biopsies have been developed; these include magnetic resonance, optical coherence tomography (OCT), and reflectance confocal microscopy (RCM).^2^

RCM allows for real-time *in vivo* visualization of the epidermis and the upper parts of the dermis at the cellular level.^3^^,^^4^ It provides information on the geometrical and topological properties of the observed tissue.^5^ It is noninvasive, thus enabling repetitive sampling of the same area without damage. This makes it a technique of choice when studying dynamic changes of the upper parts of skin over time.^6^^,^^7^ In addition, RCM allows for the quantitative study of skin cellular structures involved in the makeup of the skin barrier.^8^ Unfortunately, it is limited by the maximum depth before the signal-to-noise ratio of the image becomes too low to extract any information, but it provides information faster than microscopic analysis of a biopsy sample.

The confocal microscope was first invented by Minsky in 1957.^9^^,^^10^ Two versions are currently in use for clinical studies: a handheld *in vivo* skin imaging microscope^11^ and a wide-probe RCM.^12^ They offer a horizontal resolution of 0.5 to 1  μm and a vertical resolution (optical section thickness) of 3 to 5  μm, to a depth of 150 to 200  μm depending on the observed site.^12^

RCM is based on the collection of signals arising from light reflections at the interface of microstructures with different indices of refraction. Such microstructures are cell membranes, melanosomes, collagen fibers, lipidic layers, keratin fibers, and intracellular organelles.^13^ The closer the size of the organelle is to the wavelength of the light source and/or the higher its refractive index is compared with its surroundings, the brighter it appears.^2^

RCM is an appropriate technology to study structures *in vivo*, as the energy of the incident light, although sufficient to produce a signal, is too weak to initiate a photobiological process, hence allowing for a visualization of living cells without disturbing or changing their structure or function. By contrast, the collection of a biopsy is accompanied by local inflammatory reaction of the tissue.

**Fig. 1:**
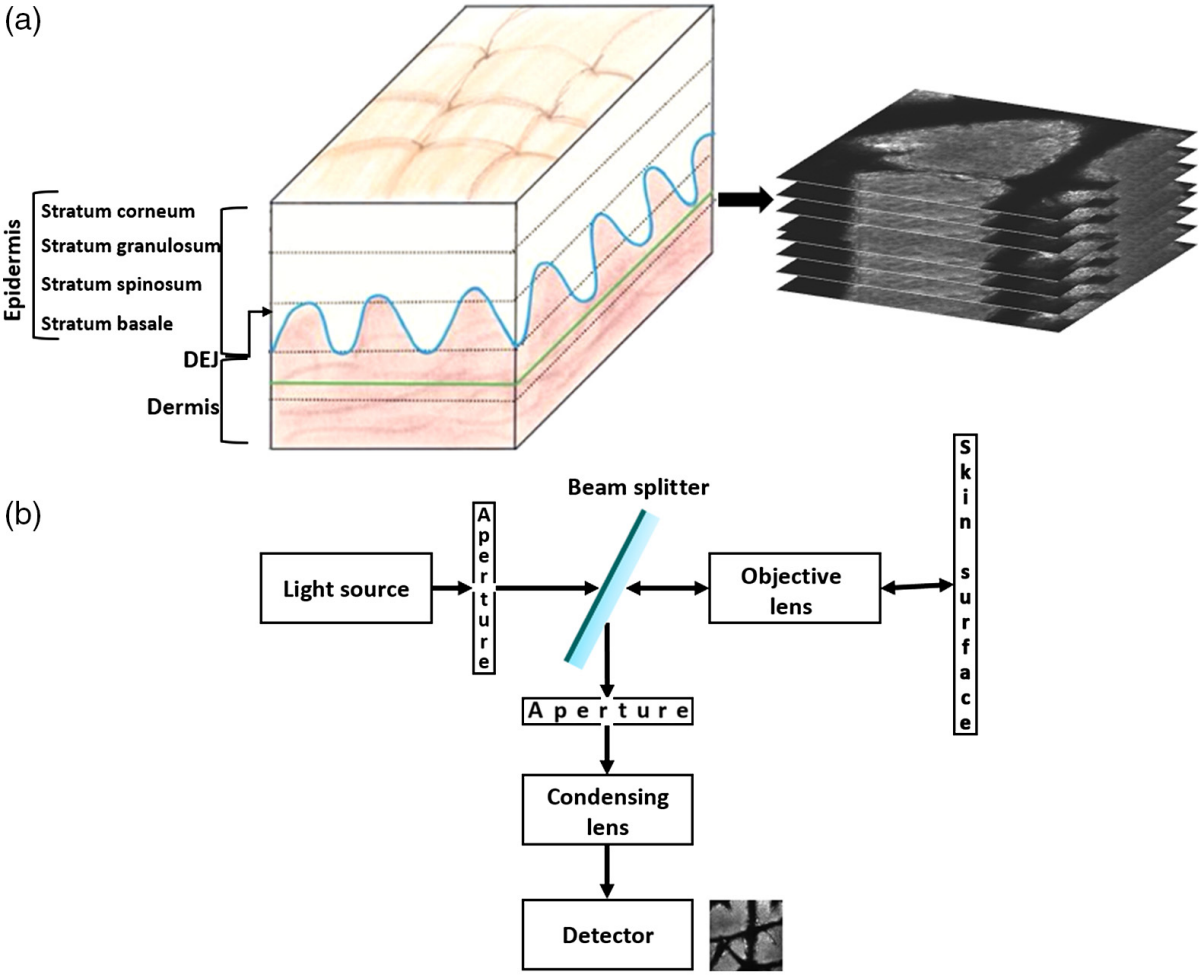
(a) RCM stacks are gray-scale images, orthogonal to the vertical sections typical of skin histology. The left panel shows an illustration of the undulating DEJ in blue. The depth limit of RCM is delineated in green, and RCM optical sections are represented with black dotted lines. The right panel shows a stack of images at sequential depths corresponding to these optical sections. (b) Diagram of an *in vivo* reflectance confocal microscope. RCM, reflectance confocal microscopy; DEJ, dermal–epidermal junction.

RCM stacks are gray-scale images acquired at sequential depths from the skin surface. Their orientation is orthogonal to the vertical sections typical in histopathology [Fig. 1(a)].

An *in vivo* reflectance confocal microscope is composed of a light source (commonly a near-infrared laser), pinhole apertures, lenses, a detector, and a mechanism for beam-scanning^14^ (Fig. 1). In the reflectance configuration, the objective lens is used to focus the illumination to a spot on the sample and to collect the reflectance signal. The detection pinhole only passes light reflected from the illuminated point in the sample to the detector and significantly rejects scattered light. By scanning the illumination spot on the sample, an image is reconstructed.

## 2 RCM Imaging in Skin Research

### 2.1 RCM Imaging of the Epidermis

The epidermis is an avascular keratinized stratified squamous epithelium generally comprising four distinct layers. From superficial to deepest, these layers are called stratum corneum (SC), stratum granulosum, stratum spinosum, and stratum basale. In the soles and palms, a thicker epidermis is observed, with an additional fifth layer between the cornified and granular layer called the stratum lucidum.

The majority of cells in all layers below the SC are referred to as keratinocytes, named due to their involvement in manufacturing and storing of keratin intermediate filaments. In contrast to the viable keratinocytes, SC is made of dead but enzymatically active keratinocytes called corneocytes.^15^ Throughout the lifetime of a person, these cells are shed and replaced by others from the lower layers. The process starts in the basal layer, where cells are continuously produced (by stem cells and transient amplifying cells), lose their attachment to the basal membrane, and migrate toward the upper layers, while undergoing differentiation toward final cell death in a process called cornification.

RCM can be used to observe the epidermal layers, the dermal–epidermal junction (DEJ), and the upper layers of the dermis,^7^ thus allowing for the computation of several quantitative descriptors of skin structure, such as keratinocyte density, number of basal keratinocytes around each dermal papilla, length of DEJ, and circumference of dermal papillae. Measuring such parameters on RCM images enables the quantitative study of skin structures and their evolution over time, for example, as a response to different stimuli. In addition to the geometrical parameters, we can also extract information about the topological organization of the epithelium, for example, the distribution of the number of nearest neighbors to each cell, an important factor in determining molecular exchange rates between neighboring cells.^5^

**Fig. 2:**
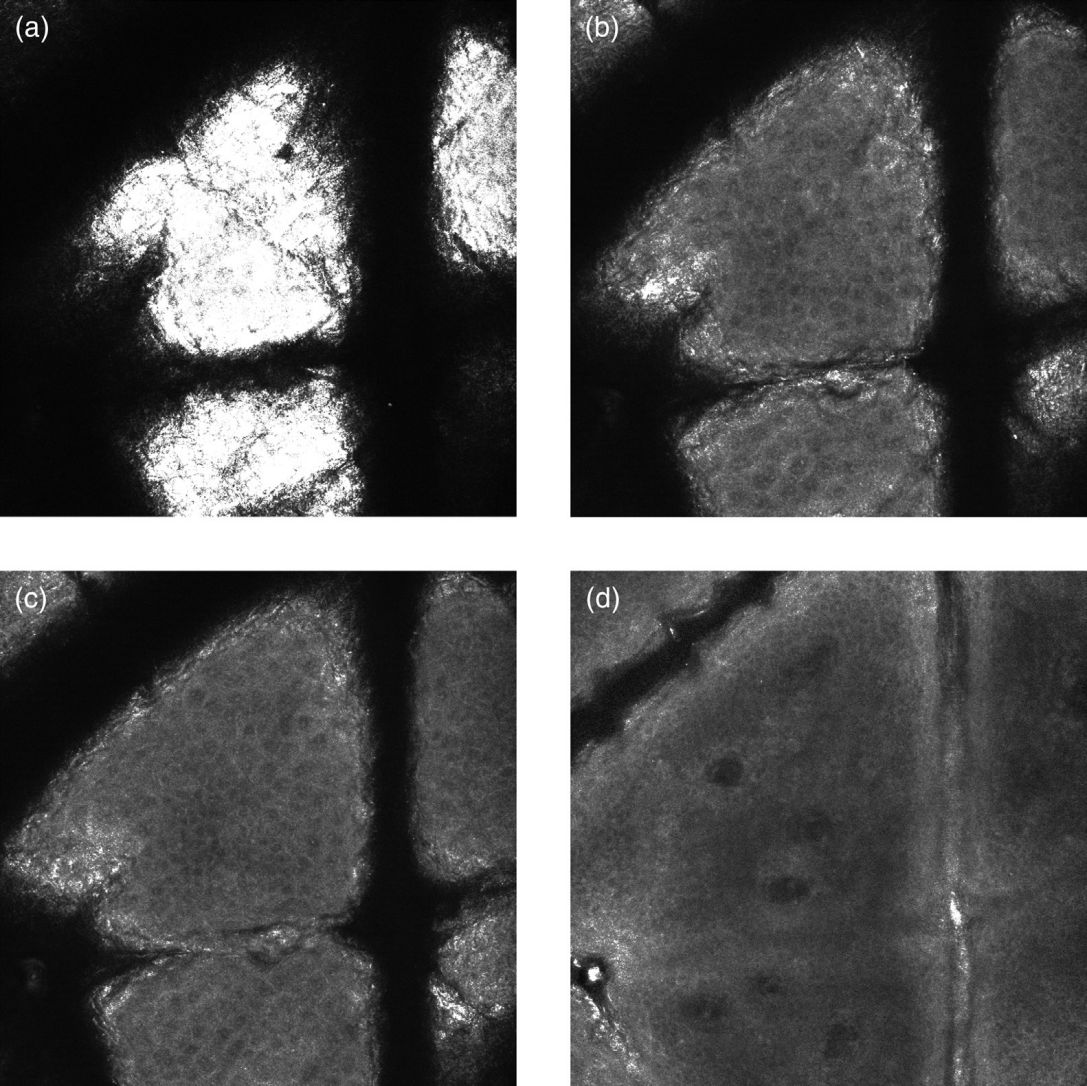
Representative RCM images of a minimally pigmented person acquired at depths corresponding to the (a) SC, (b) stratum granulosum, (c) stratum spinosum, and (d) stratum basale. RCM, reflectance confocal microscopy. Contrast was adjusted for clearer images.

The top slices of RCM stacks represent the SC, which appears as large bright areas forming islands surrounded by dark empty areas [Fig. 2(a)]. These dark areas are due to grooves called skin microrelief lines,^3^ whereas the bright signal in the island structure is due to the high reflectance of keratin. The cells are anucleated dead corneocytes, made primarily of aggregated keratin filaments embedded in a lipid matrix,^15^ polygonal in shape, and 10 to 30  μm in size.^2^

SC thickness is an important factor involved in skin barrier function.^8^^,^^15^ The thicker the SC is, the more difficult it is for a noxious substance to penetrate into the viable parts of the epidermis (or equivalently for water to transvers the epidermis and evaporate, potentially leading to tissue desiccation). It is 12 to 208  μm thick depending on the body site.^16^ Moreover, corneocytes provide a mechanical strength to the skin surface and are involved in protecting the lower layers against UV radiation, and the lipid matrix is important in maintaining skin permeability.^17^^–^^19^ SC thickness can be calculated by the depth difference of the uppermost and lowest optical sections that contain SC structures.

**Fig. 3:**
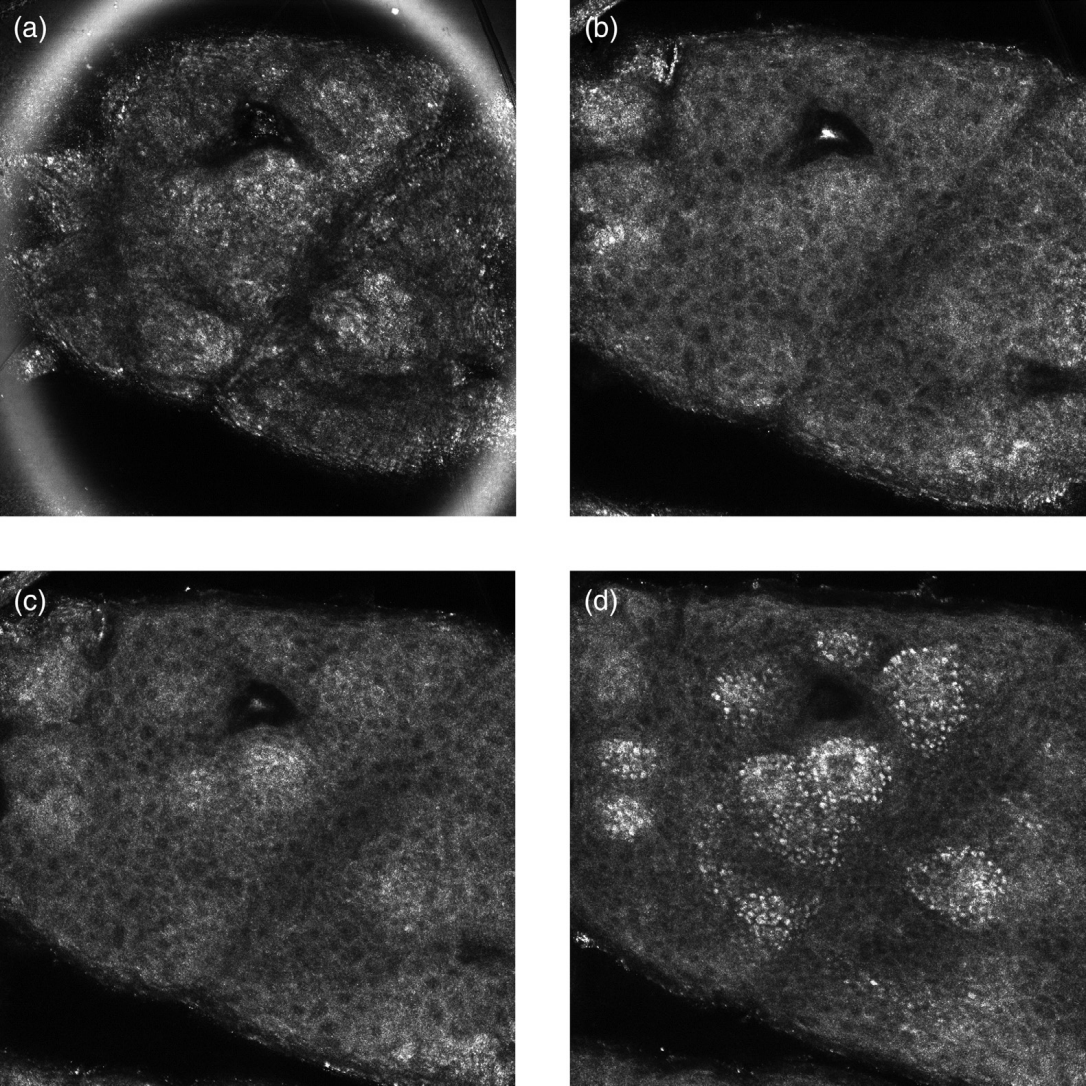
Representative RCM images of a heavily pigmented skin acquired at depths corresponding to the (a) SC, (b) stratum granulosum, (c) stratum spinosum, and (d) stratum basale. RCM, reflectance confocal microscopy.

The stratum granulosum [Fig. 2(b)] and stratum spinosum [Fig. 2(c)] are the second and third layers in the epidermis from the skin surface, respectively. They are composed of keratinocytes arranged in a honeycomb pattern in minimally pigmented skin and a cobblestone pattern in heavily pigmented skin^7^ [Figs. 3(b) and 3(c)]. In minimally pigmented skin, the cells are characterized by a dark center and grainy cytoplasm due to organelles and microstructures^6^ and are surrounded by bright membranes.^2^^,^^7^ In heavily pigmented skin types, due to the high melanin-content in melanosomes, which gives a strong reflectance signal, we observe bright keratinocytes separated by a dark contour.^20^ Viable keratinocytes are found at depths of 20 to 100  μm^21^ and are about 10 to 15  μm in size.^2^ Cells are typically larger in the granular layer than in the spinous layer,^5^ where they have a higher density. Indeed, as the keratinocytes further differentiate while climbing toward the surface, they get wider and flatter.

Toward the basal layer of the epidermis, the cells appear similar in shape but smaller in size compared with the two previous layers [Fig. 2(d)]. In contrast to the other layers, the basal keratinocytes make a monolayer. These cells are precursors of the keratinocytes in the upper layers and appear brighter than them due to the presence of melanin, which has a high reflectance.^22^^,^^23^ Melanin is made by melanocytes scattered through the basal layer and then transferred to the keratinocytes.^24^ The cells of this layer are adherent to a collagenous membrane that separates the epidermis from the dermis called the basement membrane.

The thickness of the viable epidermis is calculated as the depth difference between the optical sections at which we observe discernable viable keratinocytes in the stratum granulosum and that at which the top of the DEJ appears in the stratum basale.

The undulating DEJ [Fig. 1(a)] separates the epidermis and dermis and is located at 50 to 100  μm below the skin surface.

**Fig. 4:**
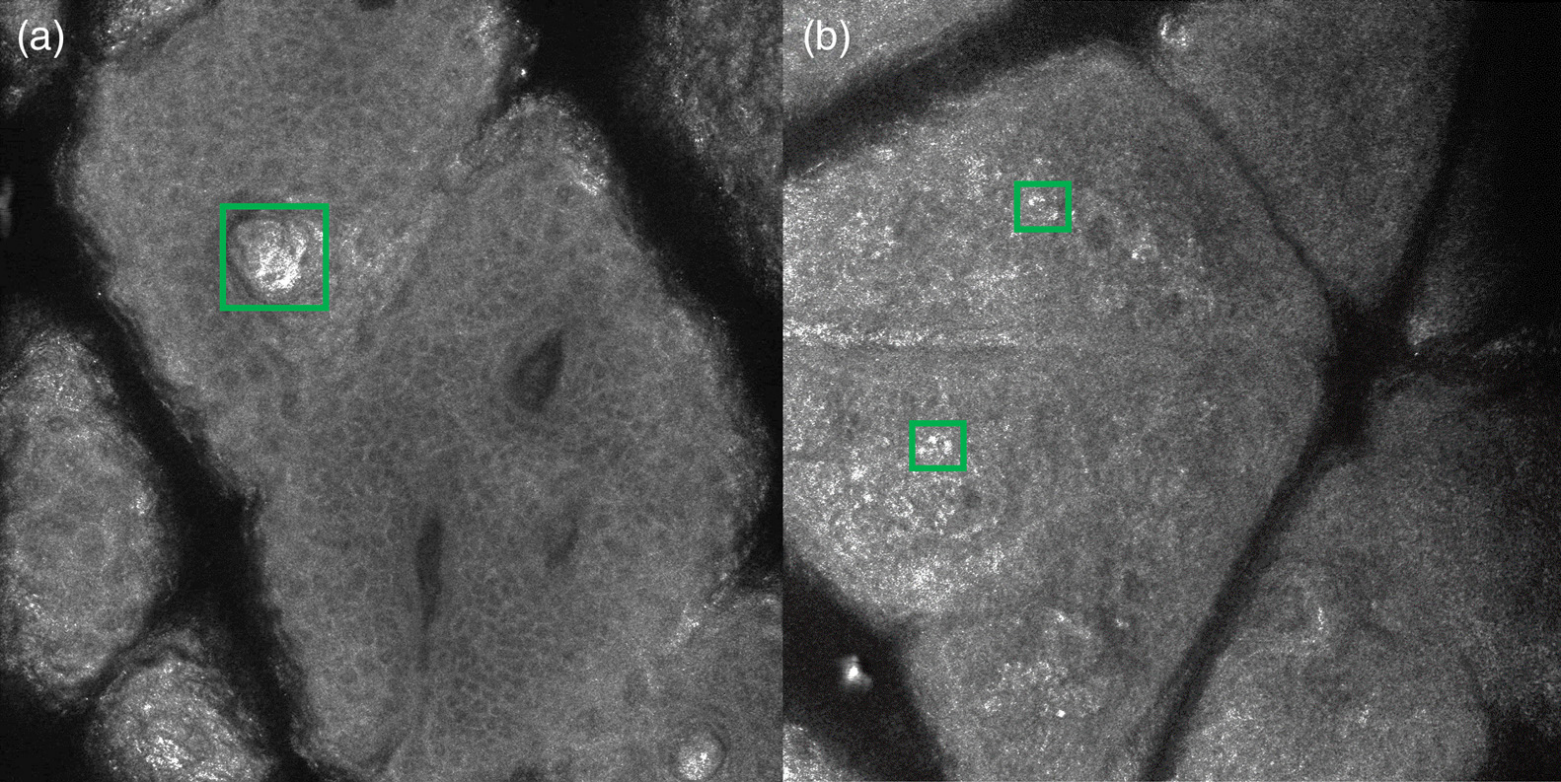
RCM images with bright spots due to (a) keratin in hair shafts and (b) clustered keratinocytes. RCM, reflectance confocal microscopy. Contrast was adjusted for clearer images.

Sometimes, bright areas can be observed on RCM images at various layers. They may arise from the keratin in hair shafts [Fig. 4(a)] or from clustered keratinocytes, also called mottled pigmentation [Fig. 4(b)].^1^

### 2.2 Clinical Study Findings Using RCM Images

#### 2.2.1 Skin maturation and aging

Skin function and structure change throughout our lifetime,^25^^,^^26^ whether this refers to skin maturation during childhood or to skin aging during adulthood. RCM allows us to visualize such changes by imaging the skin of different age groups.

Infant skin is structurally different from that of an adult starting from the appearance of the skin surface to the thickness of the epidermal layers and the extracellular matrix structures in the dermis. On the surface, infant skin has thinner more abundant microrelief lines compared with adult skin.^5^^,^^8^ This is an important point because the spaces in the microrelief lines may act as reservoirs of topically applied substances, affecting their permeability kinetics. In addition, when comparing RCM images of adults and children, we observe that the SC is 30% thinner in children and the suprapapillary epidermis is 20% thinner.^8^^,^^27^

The structural differences between infant and adult epidermis translate into functional differences, e.g., when measuring trans-epidermal water loss rates, we notice that it is significantly higher in infants and decreases throughout childhood toward the values recorded in adults.^8^

We can also observe on RCM images that, due to higher cell turnover^27^ in infant epidermis, keratinocytes and corneocytes are smaller; therefore the cell density decreases with age in both stratum granulosum and stratum spinosum.^5^ Both cell area and cell perimeter, which can be measured on RCM images, increase with age,^5^ as well as overall epidermal thickness and individual layer thickness.^27^

Changes in skin structure do not stop in adulthood as has been documented and quantified in studies using RCM.^28^ Early signs of skin aging can be observed,^1^ and the effects of cosmetic products can be evaluated. Indeed, in subjects over 70 years of age, Longo et al. observed more irregularly shaped keratinocytes, an increased compactness of collagen fibers under the DEJ, a thinning of the epidermis, and an increase in the presence of clustered keratinocytes appearing as bright spots in RCM images.^1^ In addition, although the overall epidermal thickness decreases after 50 years of age, the SC thickness increases.^29^ In addition, the number of dermal papillae decreases in aged skin,^30^ as does their size, compared with young skin.^29^

These changes in skin epidermal structure related to age become more obvious in sun-exposed areas where physiological aging related to biochemical processes is accelerated by photo-aging caused by continuous exposure to UV radiation. We can therefore connect structural changes of the epidermis observed with RCM to clinical manifestations of aging, e.g., wrinkles, thinning of the skin, hyperpigmentation spots, and loss of elasticity.

As previously mentioned, RCM can be used in studies requiring repeated assessment of skin structures in healthy as well as diseased skin.

#### 2.2.2 Photoaging

A known risk factor of skin damage, premature skin aging, and even skin cancer is exposure to UV radiations.^31^ Because RCM allows us to visualize noninvasively different elements of skin structure at different epidermal layers, it makes the longitudinal studies of skin responses to stimuli such as UV irradiation possible. Therefore, RCM is a reliable technology to assess solar damage.^32^ Using RCM images, it has been observed that the SC may appear brighter in sun-exposed areas than in sun-protected areas.

Overall epidermal thickness and keratinocyte density are greater in sun exposed areas and on the face,^6^ and their honeycomb pattern organization is often disturbed in sun-damaged regions.^32^

#### 2.2.3 Skin inflammatory diseases

RCM has been used to study inflammatory skin conditions, such as psoriasis^33^ and allergic contact dermatitis,^17^ to evaluate descriptive features of skin inflammation *in vivo* noninvasively, and to support diagnosis.

Psoriasis is characterized by a thickening of the SC and viable epidermis; both features are quantifiable by RCM. RCM has been used in patients with psoriasis to document thinning of the granular layer,^2^ increase in the number and size of dermal papillae, and increase in keratinocytes size and brightness.

Diagnosis of allergic contact dermatitis can be guided by RCM. Some of its characteristics observable in RCM are disrupted SC, vasodilatation, increased epidermal thickness, and detached corneocytes.^17^^,^^34^

RCM is limited by the maximum depth before the signal-to-noise ratio of the image becomes too low to extract any information. Nevertheless, it provides information faster than microscopic analysis of a biopsy sample and therefore can be integrated as an initial step in clinical diagnosis,^35^ e.g., guiding biopsies and determining lesion borders.^36^

#### 2.2.4 Skin cancer

Finally, 18 RCM features have been identified as useful in skin cancer diagnosis; two of them, atypical cells, and DEJ disarray, are specific for malignant melanoma (MM) diagnosis.^37^ With increasing MM incidence in Europe, RCM-aided MM diagnosis can help with early detection and thus increase survival rates.^38^ Using RCM in MM diagnosis may also reduce the number of unnecessary biopsies and benign skin lesion biopsies.^12^

In addition to diagnosis, RCM can also be used in the examination of MM and nonmelanocytic skin tumors. Indeed, the large field of view provided by RCM can be used to determine the lesion margins as it can cover a much larger area than classical histopathological approaches, such as biopsies.^2^^,^^12^ Therefore, it enables the monitoring of tumor growth and response to nonsurgical treatment and potentially guides its surgical excision.

#### 2.2.5 Cosmetology

Biopsies and other invasive methods are rarely used in the study of cosmetic product effect on skin due to ethical considerations. RCM provides a noninvasive alternative that can help to link the changes in structure to changes in appearance, e.g., aging. RCM is useful for assessing the impact of topical formulations on the cellular structure of the skin, e.g., retinoic acid and retinol,^39^ and to measure the *in vivo* kinetics of the skin after application of moisturizers,^40^^–^^43^ e.g., by measuring epidermis thickness and width of skin folds. Assessment of moisturizer photoprotection efficacy^44^ and cleanser efficacy^45^^,^^46^ is also possible with RCM.

### 2.3 RCM Limitations

All of the previously discussed studies are limited by the maximum depth for RCM imaging, which varies depending on the observed tissue type. RCM use is limited to the epidermis and upper layer of the dermis. Although refractive microstructures are abundant in deeper dermis (e.g., collagen fibers, inflammatory cells), light intensity and coherence drop exponentially with depth. In addition, most studies to date require manual analysis of RCM images, which calls for training that takes 4 to 6 months.^12^ The process is tedious and time-consuming and is subject to human error and interexpert variability. Feature extraction could be facilitated by automated image analysis methods. RCM use could then become more widespread^47^ in skin research, training time could be reduced, and image analysis could be standardized.

## 3 Computational Methods in RCM

### 3.1 Automatic Identification of Epidermal Layers

Attempts at automatically labeling the four epidermal layers have been made using machine learning approaches.^48^^–^^52^ The maximum accuracy obtained by these algorithms, i.e., percentage of correctly identified layers against ground truth, was reported to be 88%.^48^

**Fig. 5:**
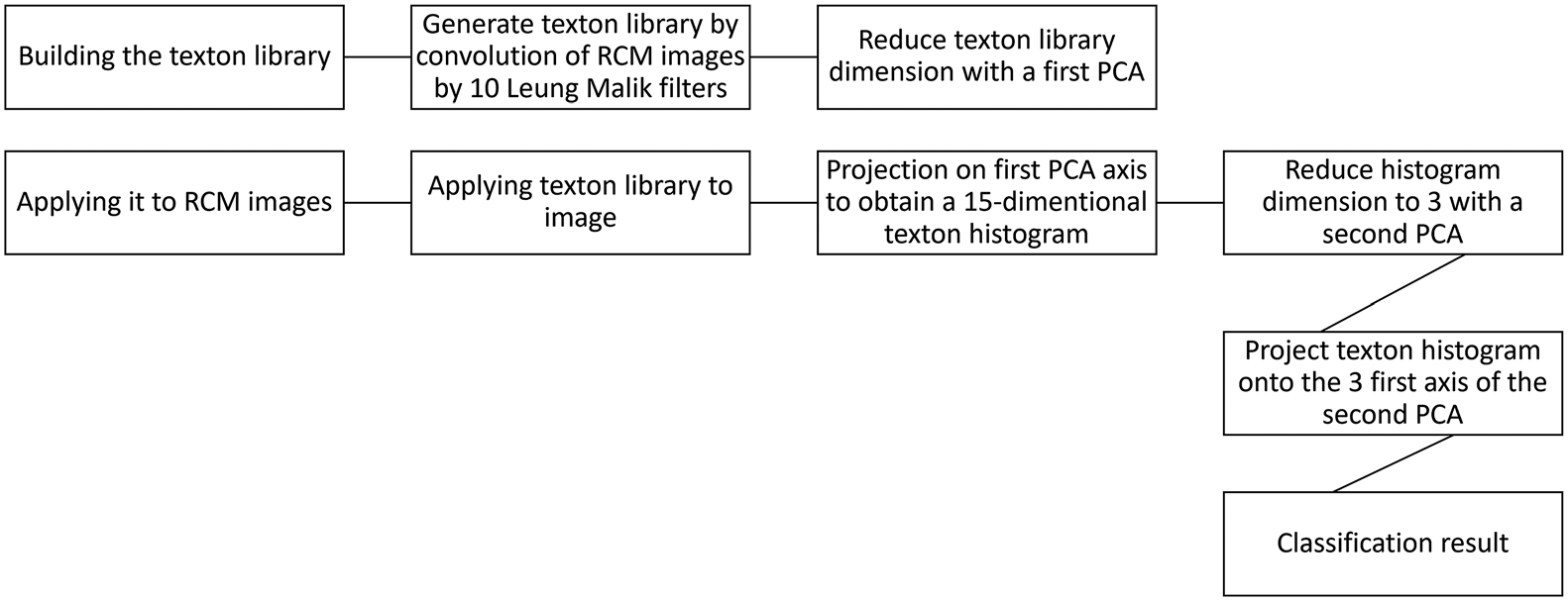
Diagram of epidermal layer classification used by Somoza et al.^51^

Somoza et al.^51^ used an unsupervised texton-based approach to achieve 54% classification accuracy. To do so, they generated a library of microstructures called textons by convolving the RCM images by 10 of the Leung–Malik filters, which matched the size of a keratinocyte. They then applied principal components analysis (PCA) to the generated filter-space to reduce the texton-space dimensionality to three, followed by a K-means clustering with 15 clusters. This texton library was applied to test RCM images, and the results were projected on the three PCA axes. Each pixel on the RCM images was classified as one the 15 textons, based on Euclidian distance, and represented as a 15-dimentional texton histogram. This histogram dimension was reduced to three by applying a second PCA. Finally, by applying K-means clustering with five clusters, for the four layers of the epidermis and the dermis, they obtained the classification of each pixel (Fig. 5).

This texton-based approach could be adapted for other classification problems, such as assessing the impact of a treatment on different skin diseases or studying skin maturation and aging. This could be achieved by extending the texton library to include more features. This method, however, could also be improved by including higher level information and features. Indeed, it does not include cellular characteristics or the presence of reflective or darker surfaces that are taken into account by experts manually identifying epidermal layers on RCM images. In addition, this was only a pilot study conducted on three stacks, and it considered that each image contains only a single epidermal layer, which is often not true.

Hames et al.^50^ used a bag of features approach to classify RCM images into four categories: SC, viable epidermis, DEJ, and papillary dermis. They established four features, inspired by prior knowledge of RCM images: (1)  the presence of a visible honeycomb pattern of viable keratinocytes indicative of the viable epidermis, (2) the presence of bands indicative of basal cell/dermal papillae indicative of the DEJ, (3) the absence of stratum basale features, and (4) visible papillae indicative of the papillary dermis. Any image before the first visible viable keratinocyte is considered to belong to the SC. Using these features, they built a feature dictionary from small image patches, which they used to represent each test image as the histogram of counts of visible features, and then they classified this histogram with an L1 regularized logistic regression as one of the four categories. They obtained a classification accuracy between 62.9% and 95.6% depending on epidermal layer, body site, and phenotype. Not all phenotypes were included, nor were all of the body sites, and the study did not include diseased skin. The method assumed the presence of a single epidermal layer per image, which is rarely true. In addition, there is no clear pattern explaining the differences in accuracies. Finally, this method does not take advantage of the three-dimensional (3-D) organization of skin to improve the results.

Kaur et al.^49^ developed a hybrid deep learning approach for classification of RCM imagers in five categories: the four epidermal layers and the dermis. First, each RCM image was convoluted by a 48-filter bank. Then using a prebuilt texton library, each pixel was represented with a patch centered around it and labeled. Each pixel was then associated to its eight nearest neighbors; thus, each image was represented by eight texton maps. These maps were pooled together by weight while the dark pixels were ignored. Finally, the obtained histograms for each image in the training set were used to train a convolutional neural network (CNN), the parameters of which were determined empirically. They achieved 82% accuracy but only tested the algorithm on three RCM image stacks. In addition, the results are limited by the features in the texton library. Using a multiresolution, multiorientation filter bank to build the library does give more features than if they were determined manually, but it complicates the interpretation of each feature.

Finally, Bozkurt et al. proposed a method to automatically classify RCM images into epidermal layers based on recurrent CNN.^48^ They introduced a mechanism called a Toeplitz structure that helps in the interpretation of the model by informing on which image the model’s decision was based. This model is an encoder–decoder, with bidirectional recurrent units and Inception V3^53^ networks. This approach achieved 88% accuracy in classifying RCM images into epidermis, DEJ, and dermis. This approach was tested on a much bigger dataset than the methods described above. Moreover, it includes higher level information by considering three surrounding images to make a prediction, which is frequently done when manually identifying epidermal layers, but using a CNN leads to a loss of feature interpretability.

Overall, neural network-based approaches^48^^,^^49^ were more successful in correctly classifying images to epidermal layers than algorithms based on texture analysis,^50^^,^^51^ but comparison between methods is not straightforward as the datasets used for each method vary in size, from 15 to 504 stacks, i.e., from 1500 to 21,412 images. The methods also vary in types of included samples, with some containing only normal healthy skin and others also including lesional skin. Finally, not all RCM stacks were taken in the same sites, but all were captured using a VivaScope 1500. Table 1 summarizes the methods described for automated epidermal layer classification.

**Table 1:** Epidermal layer classification algorithms in the literature.

Reference	Year	Method	Images database	Accuracy	Sensitivity	Specificity
51	2014	Unsupervised texton-based approach	Testing: three stacks of adult males	54%	0.53	0.9
			Type of sample: normal			
			Site: volar forearm			
50	2016	Bag of features and logistic regression	304 stacks (54 volunteers, age 20 to 30 and 50 to 70)	86%	0.84	0.92
			Type of sample: normal			
			Phototypes: 1 to 4			
			Site: dorsal and volar forearm			
49	2016	Hybrid deep learning	15 stacks (1500 images)	82%	0.72	0.96
			Training: 12 stacks			
			Testing: 3 stacks			
			Type of sample: normal			
			Site: –			
48	2017	Recurrent CNN	Training: 245 stacks	88%	0.87	0.94
			Testing: 61 stacks			
			Validation: 198 stacks			
			Type of sample: normal, benign melanocytic, and diseased			
			Site: arms, legs, torso			

### 3.2 Automatic Identification of the DEJ

The DEJ plays a fundamental role in wound healing and molecular exchanges in the skin. Skin cancer lesions are often characterized by DEJ structural changes. In addition to applications related to skin cancer, rapid localization of DEJ would be helpful in the study of healthy skin structure, for example, related to aging. Such approaches have been tested to delimitate the DEJ in RCM images, with varying levels of accuracy. Indeed, the DEJ is harder to delineate in lighter pigmented skin due to the lack of strong melanin signal. Some researchers have considered the DEJ identification to be a segmentation problem, whereas others look at it as a classification problem.

Kurugol et al.^52^ achieved 88% accuracy in more pigmented skins and 60% accuracy in minimally pigmented skins, with an average distance from the expert labels of 7.9 and 8.0  μm, respectively, using a bag of features approach with support vector machine (SVM) algorithms. Indeed, Kurugol et al.^52^ started by applying to each image a skin type detection algorithm based on the detection of bright cells in the stratum basale, which is characteristic of heavily pigmented skin. On RCM images of heavily pigmented skin, they clearly detected the DEJ, but for images of minimally pigmented skin, where melanin contrast is lower, a transition zone between dermis and epidermis was detected instead.

**Fig. 6:**
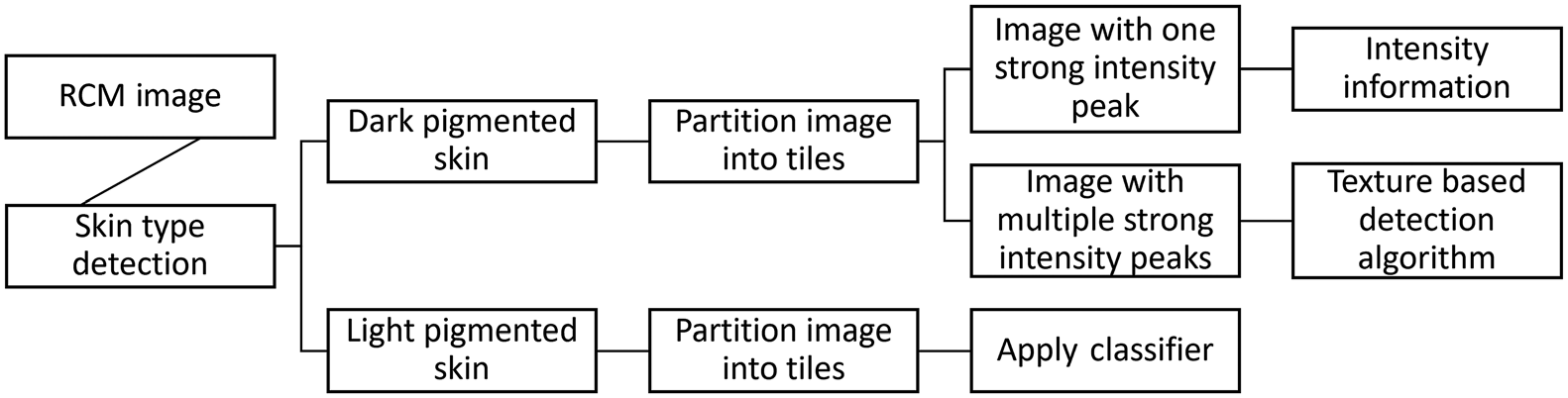
Diagram of DEJ delineation used by Kurugol et al.^52^ DEJ, dermal–epidermal junction.

For RCM images of heavily pigmented skin, where the image contained bright basal cells and had one strong intensity peak, they relied on intensity information to determine DEJ. If the image had more than one intensity peak, then they used a texture-based detection algorithm. For minimally pigmented skin, Kurugol et al. applied an SVM classifier trained on features from manually labeled images. These features were automatically selected from a list of 170 computed parameters and were the most discriminative and less redundant in the training set (Fig. 6). This approach showed better results on heavily pigmented than on minimally pigmented skin. On minimally pigmented skin, the approach failed to identify the DEJ where wrinkles are present, limiting its use. In addition, Kurugol et al. mentioned a cross-expert correlation of 81% in DEJ delineation on minimally pigmented skin, highlighting the difficulty of this task but also showing that their automated method is not on par with manual identification of the DEJ by experts.

**Fig. 7:**
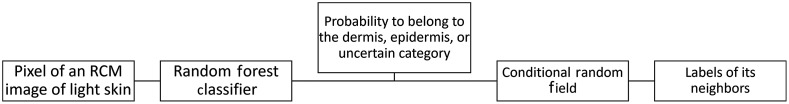
Diagram of DEJ delineation used by Robic et al.^54^^,^^55^ DEJ, dermal–epidermal junction.

Robic et al.^54^^,^^55^ used a random forest classification combined with spatial regularization based on a 3-D conditional random field^54^^,^^55^ (CRF) and achieved 54% accuracy in identifying the DEJ and 90% and 75% accuracy in identifying the epidermis and dermis, respectively. For each pixel in an RCM image of light pigmented skin, they predicted the probability of it belonging to one of three categories, i.e., dermis, epidermis, and uncertain, with a random forest classifier. These results were fed to the CRF to predict the labels of the pixel neighbors by imposing the transition order of the epidermal layers (Fig. 7). This method was not tested on RCM images of heavily pigmented skin, but it performed on par with state-of-the-art methods^48^^–^^50^^,^^52^ on minimally pigmented skin. It took into account the possibility of different layers per image, which other methods do not, as they performed classification at the image level and not at the pixel level.

Bozkurt et al. proposed a method^47^ to automatically delineate DEJ based on recurrent CNN with attention to aid cancer diagnosis. This approach achieved 88% accuracy. It is based on the knowledge that skin maintains a strict sequential ordering: epidermis, DEJ, and dermis so recurrent CNN are appropriate to automatically identify epidermal layers on RCM images. Bozkurt et al. first trained a deep CNN and then augmented it with recurrent layers, so the model would take into consideration information from the image and its surrounding slices. The CNN model used was an Inception v3 network.^53^ This method was tested on a much bigger dataset than other approaches, which allowed the authors to take into consideration the dependencies between sections, i.e., order of the layers, accounting for the 3-D organization of the skin, which helps circumvent the difficulty of identifying the DEJ by giving information on the DEJ shape across different images. However, the complexity of the model and the size of the training set hindered the training computational performance.

Finally, a Poisson point process^56^ approach managed to identify the DEJ with an average error of 5.4  μm in heavily pigmented skin and of 12.1  μm in light pigmented skin. It is an unsupervised generative framework that segments and detects complex objects, based on prior knowledge of the shape. Here, the DEJ was modeled as a succession of an unknown number of hills at random locations determined by a marked Poisson process. This method is based on the assumption that prior shape information improves object detection and thus provides an explicit model of the boundary, whereas other methods rely on extracted features. However, it requires parametrization of the Poisson point process based on prior knowledge, which in turn requires detailed labeling of the images. Therefore, the parameters in this approach can easily be interpreted, which is often not the case with deep learning-based approaches.^47^^,^^55^ On the other hand, this method relies on a small number of structural and physiological features that are meaningful to the expert, which makes it reliable but could lead to a loss of information related to other characteristics, sometimes even determined by the machine but unknown to the expert.

All of the methods used images acquired with a VivaScope 1500 microscope, but not all images were captured at the same body site. Table 2 summarizes the methods described for DEJ delineation.

**Table 2:** DEJ delineation algorithms in the literature.

Reference	Year	Method	Images database	On highly pigmented skin		On minimally pigmented skin	
				Accuracy (correctly classified image)	Average distance from the expert labeled boundaries	Accuracy (correctly classified image)	Average distance from the expert labeled boundaries
52	2012	Bag of features approach and SVMs	15 light pigmented skin and 15 heavily pigmented skin stacks	88%	7.9 μm	60%	8.0 μm
			Type of sample: normal				
			Site: forearm				
56	2017	Poisson point process	Testing: 15 stacks from volunteers aged 20 to 50	—	5.4 μm	—	12.1 μm
			Type of sample: normal				
			Site: arm and trunk				
55	2019	Random forest classification with spatial regularization based on a 3-D CRF	23 stacks from 15 volunteers	Study did not include heavily pigmented skin images	—	54%	Was not measured in the study
			Phototypes: 1 to 3				
			Type of sample: normal				
			Site: cheek				
47	2021	CNN augmented with recurrent neural network	Training: 245 stacks	Study did not include heavily pigmented skin images	—	88%	Was not measured in the study
			Testing: 61 stacks				
			Validation: 198 stacks				
			Type of sample: benign and suspicious samples				
			Site: arms, legs, torso				

DEJ, dermal–epidermal junction.

### 3.3 Automatic Identification of Pigmented Skin Lesions

Automated identification of lesions from RCM images has also been investigated. We distinguish two types of applications for these algorithms: (1) finding melanoma patterns and (2) distinguishing nonmelanocytic lesions from melanoma.

For the first application, an algorithm to identify melanocytic lesions on RCM images based on wavelet transform obtained moderate success with 55% of the melanomas and 47% of the benign nevi being correctly identified.^57^

Another approach aimed to reproduce clinician analysis of an RCM image to determine the presence of melanoma^58^ by identifying patterns in the DEJ mosaics and classifying them into melanoma or nonmelanoma with a sensitivity of 55% to 81% and a specificity of 81% to 89%. A more recent approach by Bozkurt et al.^59^ used a multiresolution convolution neural network to identify similar patterns as Kose et al.^58^ It achieved 95% average specificity and 77% average sensitivity.

For the second application of distinguishing between melanomas and nonmelanocytic lesions, Halimi et al.^60^ proposed a Bayesian model to quantify RCM images reflectivity and classify images into two categories, healthy and lentigo patients, based on their reflectivity distribution. They obtain an accuracy of 98%.

Zorgui et al.^61^ obtained similar results, with an accuracy of 98% with a CNN. The CNN was trained on normalized resized RCM images with a pretrained Inception V3 model.^53^ Transfer learning was then used to apply the model to skin RCM images.^62^ Table 3 summarizes the methods described for skin lesions identification.

**Table 3:** Some skin lesions identification algorithms in the literature.

Reference	Year	Lesion	Method	Result
57	2011	Melanocytic lesions	Wavelet transform with SVM	55% accuracy for melanomas detection
				47% accuracy for benign melanocytic nevi detection
63	2010	Superficial spreading melanoma versus nevi	Pattern recognition algorithm	100% accuracy in small pilot study
58	2016	Mosaics of the DEJ	SVM	55% to 81% sensitivity, 81% to 89% specificity
59	2018	Melanomas	CNN	77% average sensitivity, 95% average specificity
60	2017	Lentigo	Bayesian model	98%, accuracy, 96% sensitivity, 100% specificity
61	2020	Lentigo	CNN	98% accuracy, 96% sensitivity, 100% specificity
59	2018	Morphological pattern	Nested U-net	73% accuracy

Finally, Bozkurt et al.^59^ proposed a CNN inspired from the U-net^64^ architecture to identify six classes: nonlesion, artifact, meshwork pattern, ring pattern, nested pattern, and aspecific/patternless. Indeed, this model was built on a dataset containing RCM images of both lesional and nonlesional skin. This model slides through RCM images with a sliding window with 75% overlap and applies three consecutive nested U-nets. This generates segmentations at different resolution levels. Each U-net model generates a probability map. The deepest U-net model only takes a sliding window as input, whereas the others use a concatenation of the upsampled probability map at the higher level and sliding window. This model achieved 73% classification accuracy.

The goal here is not to compare the performance of these approaches, as they do not all identify the same lesion, but to give an idea of what can be achievable with RCM images.

### 3.4 Automatic Identification of Cells

RCM individual cells provide important information in the assessment of skin health, but their manual identification is tedious, time-consuming, and subject to expert interpretability. Very few attempts have been made to automatically identify individual cells or nuclei in skin RCM images.

Harris et al.^65^ proposed a pulse coupled neural network to automatically segment nuclei in oral mucosa RCM images. Identifying the nuclear-to-cytoplasm area ratio is a useful indicator in the early diagnosis of cancer. Unfortunately, RCM images tend to be noisy and nonuniform, which makes the development of an accurate segmentation algorithm complicated.^66^^,^^67^ RCM images in which the background was removed were filtered, and a spiking cortical model^68^ was applied to them, followed by an artificial neural network classifier, which outputs a segmented image. The approach had 90% accuracy (trained using eight images and tested on 28). The small size of the training set may impact the accuracy of the model. It would be difficult to apply this approach to skin RCM images that are noisier than images of the oral mucosa and riddled with small organelles within the cytoplasm and membranes of similar shape and size than a nucleus.

Gareau^69^ attempted an automated identification of keratinocytes. An error function reflectance profile was trained on labeled RCM images and then tested on other images to identify keratinocytes coordinates. All images belong to the same stack. The obtained keratinocyte density matched prior knowledge based on manual counts. The model supposed that the keratinocytes center is darker than the rim. This assumption fails on basal cells due to brightly scattering melanosome caps over the nuclei. The method may be improved by training two separate models for the granular and spinous layers as their cells differ in size. It is also unclear how it behaves when tested on RCM images of other people of different ages, as keratinocytes size changes with age.

In all models described above, results differed between minimally and heavily pigmented skin or were not tested in both cases. Any algorithm developed for the analysis of RCM images should be tested on skin types with various degrees of pigmentation.

### 3.5 Discussion

Various attempts have been made at automating the analysis of RCM images in dermatology, from automatic epidermal layer classification and DEJ identification to lesion detection and cell identification. The published work to date shows that there is high potential in the application of machine learning and/or image analysis algorithms to RCM images.

The presented algorithms have various levels of success and accuracy, but a direct comparison is difficult as used datasets varied in size and skin samples varied in sampled body site, volunteer age, and phototype. Overall, the application of computational methods in RCM is made difficult by poor image quality, high noise, and low contrast. In addition, training any supervised model requires the manual labeling of the images to obtain a ground truth, which is time-consuming and tedious and, with relative variability between experts, highlights even more the need for automation in RCM image analysis. Furthermore, the used datasets often lack in variety in terms of subject ages and skin phototypes, which introduces bias to the models and reduces their general application to all populations.

Another noninvasive *in vivo* technology used in the study of skin is OCT. OCT has been used in the study of different skin lesions, especially carcinomas and inflammatory skin diseases. OCT has a greater imaging depth than RCM of up to 2 mm, but its resolution is limited and does not allow for identification of individual cells. Attempts at automating epidermal layer classification, hair follicle identification, lesion detection, and skin inflammation have been made for OCT images, but similar to RCM, a generally accepted gold standard does not exist.

## 4 Conclusion

RCM can provide a quantitative evaluation of skin barrier physiology and how it changes due to age or responding certain stimuli, with near histological resolution. However, the study of RCM images is currently mainly done manually and therefore is tedious, time consuming, and subject to human interpretation. An automated approach to extract quantitative descriptors from confocal images would enable an easier, more reproducible, precise, and rigorous study of these images. Although attempts at the automation of descriptor extraction have been made, a globally accepted gold standard that combines all approaches and can be used by biologists and clinicians is still an open problem. Therefore, future research should focus on methods allowing for an easier translation of images into relevant quantifiable parameters and on making RCM easier, faster, and more accessible to use.

## References

[1] Longo C. et al., “Skin aging: *in vivo* microscopic assessment of epidermal and dermal changes by means of confocal microscopy,” J. Am. Acad. Dermatol. 68(3), e73–e82 (2013). JAADDB 0190-9622 10.1016/j.jaad.2011.08.021 22000768

[2] Gonzalez S. Gilaberte Y., “*In vivo* reflectance-mode confocal microscopy in clinical dermatology and cosmetology,” Int. J. Cosmet. Sci. 30, 1–17 (2008) IJCMDW 0142-5463 10.1111/j.1468-2494.2008.00406.x 18377626

[3] Rajadhyaksha M. et al., “*In vivo* confocal scanning laser microscopy of human skin II: advances in instrumentation and comparison with histology,” J. Invest. Dermatol. 113(3), 293–303 (1999) JIDEAE 0022-202X 10.1046/j.1523-1747.1999.00690.x 10469324

[4] Białek-Galas K. et al., “The use of reflectance confocal microscopy in selected inflammatory skin diseases,” Pol. J. Pathol. Official. J. Pol. Soc. Pathol. 66(2), 103–108 (2015). 10.5114/pjp.2015.53001 26247522

[5] Bensaci J. et al., “Geometrical and topological analysis of *in vivo* confocal microscopy images reveals dynamic maturation of epidermal structures during the first years of life,” J. Biomed. Opt. 20(9), 095004 (2015). JBOPFO 1083-3668 10.1117/1.JBO.20.9.095004 26359808

[6] Huzaira M. et al., “Topographic variations in normal skin, as viewed by *in vivo* reflectance confocal microscopy,” J. Invest. Dermatol. 116(6), 846–852 (2001). JIDEAE 0022-202X 10.1046/j.0022-202x.2001.01337.x 11407970

[7] Guida S. et al., “Reflectance confocal microscopy of aging skin and skin cancer,” Dermatol. Pract. Concept. 11(3), 2021068 (2021). 10.5826/dpc.1103a68 PMC8172052 34123564

[8] Stamatas G. Kollias N., “Infant skin maturation: Structural changes revealed by *in vivo* reflectance confocal microscopy and future perspectives,” in Reflectance Confocal Microscopy of Cutaneous Tumors, 2nd ed., Gonzalez S., Ed., CRC Press (2017).

[9] Minsky M., “Microscopy apparatus,” US3013467A (1961).

[10] Minsky M., “Memoir on inventing the confocal scanning microscope,” Scanning 10(4), 128–138 (1988). SCNNDF 0161-0457 10.1002/sca.4950100403

[11] Gareau D. Patel Y. Rajadhyaksha M., “Basic principles of reflectance confocal microscopy,” Reflectance Confocal Microscopy of Cutaneous Tumors, 2nd ed., Gonzalez S., Ed., pp. 1–6, CRC Press (2017).

[12] Levine A. Markowitz O., “Introduction to reflectance confocal microscopy and its use in clinical practice,” JAAD Case Rep. 4(10), 1014–1023 (2018). 10.1016/j.jdcr.2018.09.019 30456275 PMC6232695

[13] Raphael A. et al., “Reflectance confocal microscopy and aging,” in Textbook of Aging Skin, Farage M. Miller K. Maibach H., Eds., pp. 1381–1397, Springer, Berlin, Heidelberg (2017).

[14] Kollias N. Stamatas G. N., “Optical non-invasive approaches to diagnosis of skin diseases,” J. Invest. Dermatol. Symp. Proc. 7(1), 64–75 (2002). 10.1046/j.1523-1747.2002.19635.x 12518795

[15] Murphrey M. B. Miao J. H. Zito P. M., “Histology, stratum corneum,” in StatPearls, StatPearls Publishing, Treasure Island, Florida (2021). 30020671

[16] Böhling A. et al., “Comparison of the stratum corneum thickness measured *in vivo* with confocal Raman spectroscopy and confocal reflectance microscopy,” Skin Res. Technol. 20(1), 50–57 (2014). 10.1111/srt.12082 23909688

[17] Maarouf M. et al., “*In vivo* reflectance confocal microscopy: emerging role in noninvasive diagnosis and monitoring of eczematous dermatoses,” Actas Dermo-Sifiliográficas Engl. Ed. 110(8), 626–636 (2019). 10.1016/j.ad.2018.08.008 31202471

[r18] Goleva E. Berdyshev E. Leung D. Y., “Epithelial barrier repair and prevention of allergy,” J. Clin. Invest. 129(4), 1463–1474 (2019). JCINAO 0021-9738 10.1172/JCI124608 30776025 PMC6436854

[19] Egawa G., “Pathomechanism of ‘skin-originated’ allergic diseases,” Immunol. Med. 41(4), 170–176 (2018). 10.1080/25785826.2018.1540257 30632910

[20] Longo C., “Well-aging: early detection of skin aging signs,” Dermatol. Clin. 34(4), 513–518 (2016). DERAEG 1018-8665 10.1016/j.det.2016.05.014 27692457

[21] Elder D. E. et al., Lever’s Histopathology of the Skin, Lippincott Williams & Wilkins (2009).

[22] Sánchez-Mateos J. L. S. et al., “Reflectance-mode confocal microscopy in dermatological oncology,” in Lasers in Dermatology and Medicine, Nouri K., Ed., pp. 285–308, Springer, London (2012).

[23] Rajadhyaksha M. et al., “*In vivo* confocal scanning laser microscopy of human skin: melanin provides strong contrast,” J. Invest. Dermatol. 104(6), 946–952 (1995). JIDEAE 0022-202X 10.1111/1523-1747.ep12606215 7769264

[24] Raposo G. Marks M. S., “Melanosomes – dark organelles enlighten endosomal membrane transport,” Nat. Rev. Mol. Cell Biol. 8(10), 786–797 (2007). NRMCBP 1471-0072 10.1038/nrm2258 17878918 PMC2786984

[25] Stamatas G. N. et al., “Infant skin physiology and development during the first years of life: a review of recent findings based on *in vivo* studies,” Int. J. Cosmet. Sci. 33(1), 17–24 (2011). IJCMDW 0142-5463 10.1111/j.1468-2494.2010.00611.x 20807257

[26] Nikolovski J. et al., “Barrier function and water-holding and transport properties of infant stratum corneum are different from adult and continue to develop through the first year of life,” J. Invest. Dermatol. 128(7), 1728–1736 (2008). JIDEAE 0022-202X 10.1038/sj.jid.5701239 18200056

[27] Stamatas G. N. et al., “Infant skin microstructure assessed *in vivo* differs from adult skin in organization and at the cellular level,” Pediatr. Dermatol. 27(2), 125–131 (2010). 10.1111/j.1525-1470.2009.00973.x 19804498

[28] Cinotti E. et al., “Structural skin changes in elderly people investigated by reflectance confocal microscopy,” J. Eur. Acad. Dermatol. Venereol. 34(11), 2652–2658 (2020). JEAVEQ 0926-9959 10.1111/jdv.16466 32294278

[29] Kawasaki K. Yamanishi K. Yamada H., “Age-related morphometric changes of inner structures of the skin assessed by *in vivo* reflectance confocal microscopy,” Int. J. Dermatol. 54(3), 295–301 (2015). IJDEBB 1365-4362 10.1111/ijd.12220 25267556

[30] Lagarrigue S. G. et al., “*In vivo* quantification of epidermis pigmentation and dermis papilla density with reflectance confocal microscopy: variations with age and skin phototype,” Exp. Dermatol. 21(4), 281–286 (2012). EXDEEY 0906-6705 10.1111/j.1600-0625.2012.01451.x 22417304

[31] Kimlin M. G. Guo Y., “Assessing the impacts of lifetime sun exposure on skin damage and skin aging using a non-invasive method,” Sci. Total Environ. 425, 35–41 (2012). 10.1016/j.scitotenv.2012.02.080 22459885

[32] Haytoglu N. S. K. et al., “Assessment of skin photoaging with reflectance confocal microscopy,” Skin Res. Technol. 20(3), 363–372 (2014). 10.1111/srt.12127 24506234

[33] González S. et al., “Characterization of psoriasis *in vivo* by reflectance confocal microscopy,” J. Med. 30(5–6), 337–356 (1999). 10851567

[34] Astner S. González S. Gonzalez E., “Noninvasive evaluation of allergic and irritant contact dermatitis by *in vivo* reflectance confocal microscopy,” Dermat. Contact Atopic Occup. Drug 17(4), 182–191 (2006). 10.2310/6620.2006.05052 17150167

[35] Agozzino M. Gonzalez S. Ardigò M., “Reflectance confocal microscopy for inflammatory skin diseases,” Actas Dermo-Sifiliográficas Engl. Ed. 107(8), 631–639 (2016). 10.1016/j.adengl.2016.01.030 26996333

[36] Gonzalez S. et al., “Normal skin: terminology,” in Reflectance Confocal Microscopy of Cutaneous Tumors, 2nd ed., Gonzalez S., Ed., CRC Press (2017).

[37] Pellacani G. et al., “Reflectance confocal microscopy made easy: the 4 must-know key features for the diagnosis of melanoma and nonmelanoma skin cancers,” J. Am. Acad. Dermatol. 81(2), 520–526 (2019). JAADDB 0190-9622 10.1016/j.jaad.2019.03.085 30954581

[38] Stevenson A. D. et al., “Systematic review of diagnostic accuracy of reflectance confocal microscopy for melanoma diagnosis in patients with clinically equivocal skin lesions,” Dermatol. Pract. Concept. 3(4), 19–27 (2013). 10.5826/dpc.0304a05 24282659 PMC3839827

[39] Kong R. et al., “A comparative study of the effects of retinol and retinoic acid on histological, molecular, and clinical properties of human skin,” J. Cosmet. Dermatol. 15(1), 49–57 (2016). 10.1111/jocd.12193 26578346

[40] Manfredini M. et al., “Does skin hydration influence keratinocyte biology? *In vivo* evaluation of microscopic skin changes induced by moisturizers by means of reflectance confocal microscopy,” Skin Res. Technol. 19(3), 299–307 (2013). 10.1111/srt.12042 23441646

[r41] Bağcı I. S. et al., “Effects of short-term moisturizer application in different ethnic skin types: noninvasive assessment with optical coherence tomography and reflectance confocal microscopy,” Skin Pharmacol. Physiol. 31(3), 125–133 (2018) 10.1159/000486626 29539605

[r42] de Carvalho N. et al., “15321 Evaluation of a ceramide-containing lotion on skin hydration and cellular morphology assessed by reflectance confocal microscopy,” J. Am. Acad. Dermatol. 83(6), AB147 (2020). JAADDB 0190-9622 10.1016/j.jaad.2020.06.676

[43] Ruini C. et al., “*In vivo* examination of healthy human skin after short-time treatment with moisturizers using confocal Raman spectroscopy and optical coherence tomography: Preliminary observations,” Skin Res. Technol. 28(1), 119–132 (2022). 10.1111/srt.13101 34555219 PMC9907652

[44] Gomes-Neto A. et al., “Efficacy of a daily protective moisturizer with high UVB and UVA photoprotection in decreasing ultraviolet damage: evaluation by reflectance confocal microscopy,” Acta Dermatol. Venereol. 97(10), 1196–1201 (2017). 10.2340/00015555-2736 28661544

[45] Diluvio L. et al., “Clinical and confocal evaluation of avenanthramides-based daily cleansing and emollient cream in pediatric population affected by atopic dermatitis and xerosis,” G. Ital. Dermatol. Venereol. 154(1), 32–36 (2019). GIDVDZ 0392-0488 10.23736/S0392-0488.18.06002-9 30207438

[46] Meyers C. L. et al., “Near-infrared reflectance spectroscopy and multispectral imaging detect changes in skin hydration from cleansing products1,” J. Am. Acad. Dermatol. 50(3), P33 (2004). JAADDB 0190-9622 10.1016/j.jaad.2003.10.136

[47] Bozkurt A. et al., “Skin strata delineation in reflectance confocal microscopy images using recurrent convolutional networks with attention,” Sci. Rep. 11, 12576 (2021). SRCEC3 2045-2322 10.1038/s41598-021-90328-x 34131165 PMC8206415

[48] Bozkurt A. et al., “Delineation of skin strata in reflectance confocal microscopy images using recurrent convolutional networks with toeplitz attention,” arXiv171200192 Cs (2017). 10.1038/s41598-021-90328-x PMC8206415 34131165

[r49] Kaur P. et al., “Hybrid deep learning for reflectance confocal microscopy skin images,” in 23rd Int. Conf. Pattern Recognit. (ICPR), pp. 1466–1471 (2016). 10.1109/ICPR.2016.7899844

[r50] Hames S. et al., “Automated segmentation of skin strata in reflectance confocal microscopy depth stacks,” PLoS ONE 11(4), e0153208 (2016). POLNCL 1932-6203 10.1371/journal.pone.0153208 27088865 PMC4835045

[r51] Somoza E. et al., “Automatic localization of skin layers in reflectance confocal microscopy,” in Int. Conf. Image Anal. and Recognit., 22 October 2014, pp. 141–150. 10.1007/978-3-319-11755-3_16

[52] Kurugol S. et al., “Validation study of automated dermal/epidermal junction localization algorithm in reflectance confocal microscopy images of skin,” Proc. SPIE 8207, 820702 (2012). PSISDG 0277-786X 10.1117/12.909227 PMC3872972 24376908

[53] Szegedy C. et al., “Rethinking the inception architecture for computer vision,” arXiv151200567 Cs (2015).

[54] Robic J. et al., “Classification of the dermal-epidermal junction using *in-vivo* confocal microscopy,” in IEEE 14th Int. Symp. Biomed. Imaging (ISBI), pp. 252–255 (2017). 10.1109/ISBI.2017.7950513

[55] Robic J. et al., “Three-dimensional conditional random field for the dermal-epidermal junction segmentation,” J. Med. Imaging 6(2), 024003 (2019). 10.1117/1.JMI.6.2.024003 PMC6487290 31065567

[56] Ghanta S. et al., “A marked Poisson process driven latent shape model for 3D segmentation of reflectance confocal microscopy image stacks of human skin,” IEEE Trans. Image Process. 26(1), 172–184 (2017). IIPRE4 1057-7149 10.1109/TIP.2016.2615291 27723590 PMC5258843

[57] Koller S. et al., “*In vivo* reflectance confocal microscopy: automated diagnostic image analysis of melanocytic skin tumours,” J. Eur. Acad. Dermatol. Venereol. 25(5), 554–558 (2011). JEAVEQ 0926-9959 10.1111/j.1468-3083.2010.03834.x 20735518

[58] Kose K. et al., “A machine learning method for identifying morphological patterns in reflectance confocal microscopy mosaics of melanocytic skin lesions in-vivo,” Proc. SPIE 9689, 968908 (2016). PSISDG 0277-786X 10.1117/12.2212978

[59] Bozkurt A. et al., “A multiresolution convolutional neural network with partial label training for annotating reflectance confocal microscopy images of skin,” arXiv180202213 Cs (2018). 10.1007/978-3-030-00934-2_33 PMC12882772 41657661

[60] Halimi A. et al., “An unsupervised Bayesian approach for the joint reconstruction and classification of cutaneous reflectance confocal microscopy images,” in 25th Eur. Signal Process. Conf. (EUSIPCO), 1 August 2017, pp. 241–245. 10.23919/EUSIPCO.2017.8081205

[61] Zorgui S. et al., “A convolutional neural network for lentigo diagnosis,” Impact Digit. Technol. Public Health Dev. Dev. Ctries. 12157, 89–99 (2020). 10.1007/978-3-030-51517-1_8

[62] Ribani R. Marengoni M., “A survey of transfer learning for convolutional neural networks,” in 32nd SIBGRAPI Conf. Graph., Patterns and Images Tutorials (SIBGRAPI-T), pp. 47–57 (2019). 10.1109/SIBGRAPI-T.2019.00010

[63] Gareau D. et al., “Automated detection of malignant features in confocal microscopy on superficial spreading melanoma versus nevi,” J. Biomed. Opt. 15(6), 061713 (2010). JBOPFO 1083-3668 10.1117/1.3524301 21198161 PMC3036174

[64] Ronneberger O. Fischer P. Brox T., “U-Net: convolutional networks for biomedical image segmentation,” arXiv150504597 Cs (2015).

[65] Harris M. A. et al., “A pulse coupled neural network segmentation algorithm for reflectance confocal images of epithelial tissue,” PLoS ONE 10(3), e0122368 (2015). POLNCL 1932-6203 10.1371/journal.pone.0122368 25816131 PMC4376773

[66] Luck B. L. Bovik A. C. Richards-Kortum R. R., “Segmenting cervical epithelial nuclei from confocal images Gaussian Markov random fields,” in Proc. Int. Conf. Image Process. (Cat. No.03CH37429), Vol. 2, p. 1069 (2003). 10.1109/ICIP.2003.1246870

[67] Luck B. et al., “An image model and segmentation algorithm for reflectance confocal images of *in vivo* cervical tissue,” IEEE Trans. Image Process. Publ. IEEE Signal Process. Soc. 14, 1265–1276 (2005). 10.1109/TIP.2005.852460 16190463

[68] Ekblad U. Kinser J. M., “Theoretical foundation of the intersecting cortical model and its use for change detection of aircraft, cars, and nuclear explosion tests,” Signal Process. 84(7), 1131–1146 (2004). 10.1016/j.sigpro.2004.03.012

[69] Gareau D., “Automated identification of epidermal keratinocytes in reflectance confocal microscopy,” J. Biomed. Opt. 16(3), 030502 (2011). JBOPFO 1083-3668 10.1117/1.3552639 21456857 PMC3077366

